# *Aldh1l1-Cre/ER ^T2^* is expressed in unintended cell types of the salivary gland, pancreas, and spleen.

**DOI:** 10.17912/micropub.biology.000832

**Published:** 2023-05-19

**Authors:** Daniel Nemeth, Numana Luqman, Loretta Chen, Ning Quan

**Affiliations:** 1 Stiles-Nicholson Brain Institute, College of Medicine, Florida Atlantic University, Boca Raton, Florida, United States; 2 Florida Atlantic University High School, Boca Raton, Florida, United States; 3 College of Chemistry, Florida Atlantic University, Boca Raton, Florida, United States

## Abstract

The *Aldh1l1-Cre/ER ^T2^* mouse is a widely used transgenic mouse model to conditionally express Cre recombinase in astrocytes of the central nervous system. Currently, no reports show whether the Cre recombinase activity, driven by the *Aldh1l1* promoter, acts in cells outside of its intended astrocyte population. We crossed the *Aldh1l1-Cre/ER ^T2^* mouse with a TdTomato reporter mouse line, ROSA26:CAG-LSL-TdTomato, to generate a fluorescent reporter for *Aldh1l1* promoter activity. Gross anatomical observations reveal strong TdTomato expression in the spleen and exocrine glands—the salivary gland and the pancreas. We find TdTomato expression, a reporter of Cre activity, specifically targets serous cells in the parotid, submandibular, sublingual glands, and pancreas along with fibroblast-like cells within the submandibular lymph nodes and spleen. Our data indicate that the *Aldh1l1-Cre/ER ^T2^* mouse model has unintended Cre recombinase activity in exocrine glands, which may influence biological and behavioral data.

**Figure 1. Aldh1l1-Cre/ER T2 expression in the brain, salivary gland, pancreas, and spleen.**
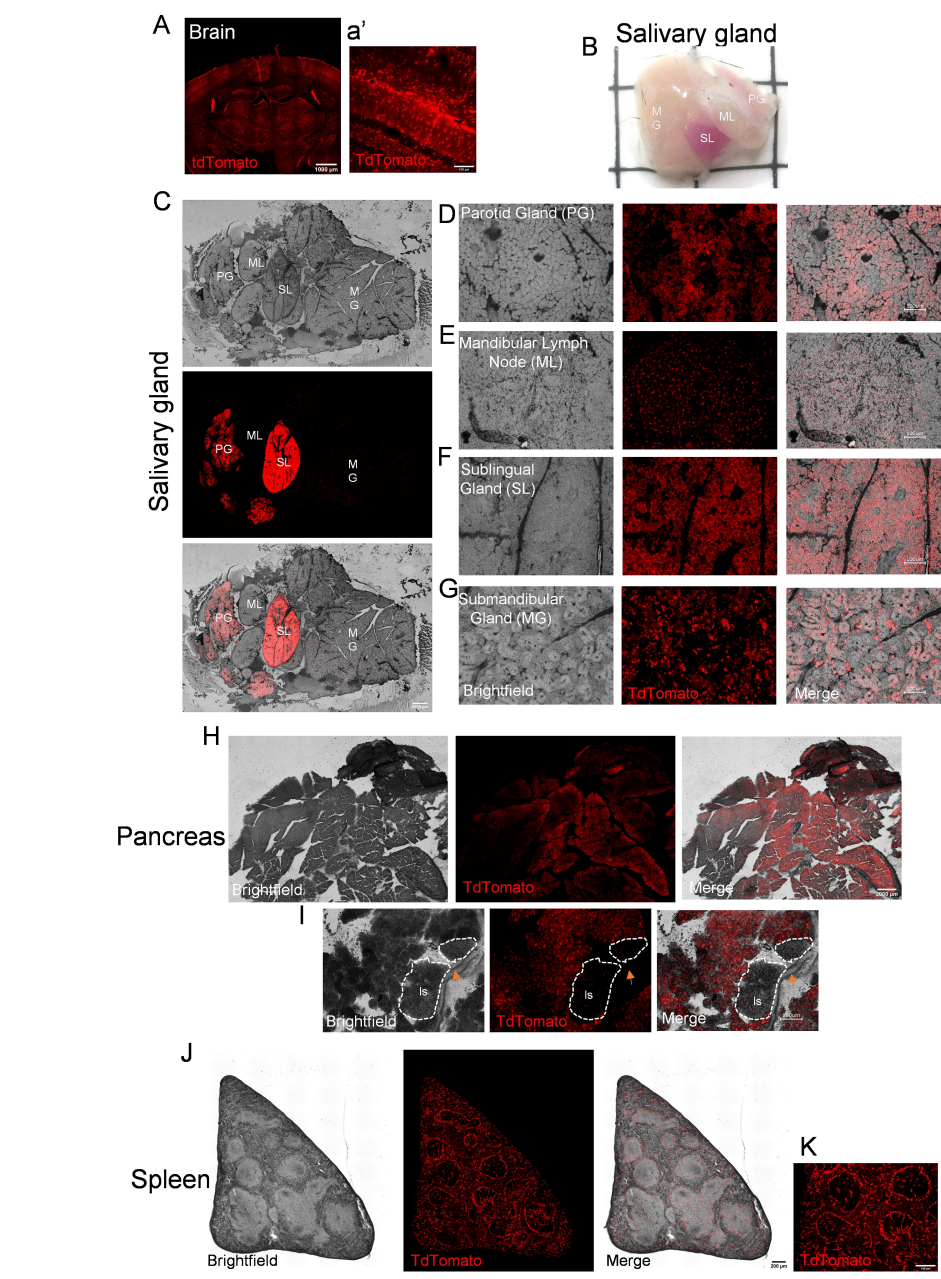
Fluorescent collage (A) and 10x fluorescent image (a’) of hippocampal brain section of *Aldh1l1-Cre/ER ^T2^* :ROSA26-LSL-TdTomato. Low magnification image of whole salivary gland. Grid lines = 5 mmx5 mm (B). Bright field, fluorescent and merged 10x collages of the salivary gland of *Aldh1l1-Cre/ER ^T2^* :ROSA26-LSL-TdTomato mice (C). 10x stack-focused brightfield, fluorescent, and merged images of the parotid gland (D), mandibular lymph node (E), sublingual gland (F), and mandibular gland (G). Bright field, fluorescent and merged 10x collages of the pancreas of *Aldh1l1-Cre/ER ^T2^* :ROSA26-LSL-TdTomato mice (H). 10x stack-focused bright field, fluorescent, and merged images of regions containing pancreatic islets (I). Bright field, fluorescent and merged 10x collages of the spleen of *Aldh1l1-Cre/ER ^T2^* :ROSA26-LSL-TdTomato mice (J). 10x stack-focused fluorescent image the spleen (K). PG=Parotid gland, ML=Mandibular lymph node, SL=Sublingual gland, MG=Submandibular gland, Is= Pancreatic islet. Dotted lines denotes region between islet and acinar cells of the pancreas. Scale bars for A = 1000 μm. Scale bars for C and H = 2000 μm. Scale bars for J = 200 μm. Scale bars for a’, D, E, F, G, I, and K = 100 μm.

## Description

The Cre-Lox recombination system has been a highly influential gene editing system and is the main method to generate conditional gene knockouts, reporters, and targeted chemo/optogenetic manipulations. Cre recombinase is a tyrosine recombinase enzyme which targets and excises DNA between a specific sequence called a LoxP site (Sauer, 1987; Sauer & Henderson, 1988) . In order for DNA excision to occur, two LoxP sites must be present spanning across a region of DNA. Insertion of two LoxP sites across a region of DNA is known as “floxing”. Once Cre detects both of the LoxP sites, it ligates the DNA at each site and the DNA strands will rejoin, effectively excising the floxed DNA. Researchers utilize the Cre-Lox mechanism to create multiple types of transgenic animals like gene knockouts, reporters, and opto/chemogenetic manipulatable animals. Gene knockouts can be created by floxing crucial regions of a gene. Gene reporters or opto/chemogenetic manipulatable animals are created by floxing a stop sequence which precedes a constitutively active gene encoding a florescent molecule or opto/chemogenetic receptor (Song & Palmiter, 2018) . Further specificity can be achieved by cell-type specific promoters driving Cre expression which selects the populations of cells that express Cre. One caveat to this method is that many cell types throughout the body may utilize the same promoter. For instance, *Tie2* is a promoter which is highly expressed in the endothelial cells of blood vessels, however, it also is utilized by hematopoietic cells of the bone marrow, meaning that Cre would be expressed in both populations if under this promoter (Koni et al., 2001) . This multi-population usage of promoters is common and typically promoter usage between organs is understudied after the generation of cell-type specific Cre expressing animals.

Aldehyde dehydrogenase 1 family member L1 ( *Aldh1l1* or 10-formyltetrahydrofolate dehydrogenase) is a gene which encodes a protein that is known to regulate folate metabolism (Sharma et al., 2022) , cell motility (Oleinik et al., 2010) , and cancer (Reviewed by Krupenko & Krupenko, 2018). Recently, the promoter for the *Aldh1l1* gene has been discovered to be ubiquitously expressed by astrocytes, a glial cell of the central nervous system. Utilizing the promoter information, transgenic mice were generated to control Cre recombinase expression under the *Aldh1l1* promoter (Srinivasan et al., 2016) . Original reports suggest this Cre recombinase model is highly selective for astrocytes in the CNS; however, whether organs besides the brain utilize the *Aldh1l1* promoter was undetermined.

We crossed the BAC *Aldh1l1-Cre/ER ^T2^* mouse (JAX Strain #:031008) to a ROSA26-LSL-TdTomato (Jax Strain #:007909) mice in order to track Cre activity via TdTomato expression driven by the *Aldh1l1* promoter throughout brain and peripheral organs. From the first litter, two mice contained both the *Aldh1l1-Cre/ER ^T2^* transgene and the ROSA26-LSL-TdTomato allele and both were used for this study. Both animals were injected with intraperitoneal tamoxifen (75 mg/kg) for five days to initiate the activity of the tamoxifen-inducible Cre recombinase and subsequent TdTomato expression. Seven days later, mice were euthanized and perfusion fixed. Organs were removed and post-fixed for 24hr in 4% PFA then cryoprotected in 20% sucrose until sectioning. Within the brain, we verified that Cre activity, via Tdtomato expression, was exclusively in astrocytes via morphological characteristics ( Fig. 1A ). Upon gross examination of peripheral organs, the salivary gland was identified as a major expresser of TdTomato. The sublingual gland (SL) was bright pink and the parotid gland (PG) was dimly pink in visible color ( Fig. 1B ). Sections of salivary glands similarly reveal the SL and PG expressed the highest levels of TdTomato ( Fig. 1C ). In the PG, TdTomato expression was found in a majority of secretory cells but absent in regions within the ducts ( Fig. 1D ). In the mandibular lymph nodes (ML), TdTomato+ cells were sparse and resemble fibroblastic stromal cells and no TdTomato+ cells made up the vasculature ( Fig. 1E ). TdTomato+ cells were found in the mucous and serous cells of the sublingual gland (SL) but not within striate ducts ( Fig. 1F ). Within the submandibular gland (MG), TdTomato expression was lowest and more diffuse where TdTomato+ cells resemble secretory acini of serous and mucous cells ( Fig. 1G ). Brightfield and fluorescent collages of the pancreas reveal widespread TdTomato expression across the pancreas ( Fig. 1H ). Specifically, TdTomato expression is expressed within the acinar cells of the pancreas. Strong TdTomato expression is found in the cell nucleus and weakly throughout the cell body; however, TdTomato was sparingly found within the endocrine insulin producing beta cells of the Islet of Langerhans (Is, Fig. 1H ). Bright field and fluorescent collages of the spleen show TdTomato expression in cells surrounding the edges of the germinal centers. Few cells express TdTomato within the germinal centers of white pulp. ( Fig. 1J -K).

Due to the selectivity for astrocytes in the brain the *Aldh1l1-Cre/ER ^T2^* is still the best conditional mouse model to study astrocyte populations in the CNS. Our results show that there are multiple organs, besides the brain, that also utilize the *Aldh1l1* promoter, such as the salivary gland, lymph nodes, pancreas, and spleen. Due to the fact that observations and manipulations using this mouse line predominantly take place in the brain, the majority of studies utilizing this model may have overlooked any non-astrocytic contributions (McKee et al., 2022; Sanmarco et al., 2021; Wang et al., 2021) . Nevertheless, it is vital to understand that non-brain *Aldh1l1-Cre/ER ^T2^* activity may have unintended effects on biological processes or behavior when creating conditional genetic knockouts and conducting chemogenetic manipulations. One way to confirm astrocyte-mediated perturbations is via viral vectors which preferentially infect astrocytes (Griffin et al., 2019; O’Carroll et al., 2021). Indeed, rigorous studies have confirmed their hypotheses with the use of astrocyte-specific adeno-associated viruses that express Cre in a brain region specific manner which is a crucial control in contrast with this transgenic mouse model (O’Neil et al., 2022). Overall, our data show for the first time, that *Aldh1l1-Cre/ER ^T2^* activity has off-target effects in many peripheral organs. More examination of other organs and circulating blood cells is required to determine the widespread usage of *Aldh1l1* throughout the periphery.

## Methods

Mice:

A male *Aldh1l1-Cre/ER ^T2^* transgenic mouse line (JAX Strain #: 031008) and a female ROSA26-LSL-TdTomato reporter mouse line (JAX Strain #: 007909) were crossed to generate the experimental mice. The mice were housed under standard laboratory conditions with 12-hour light-dark cycles (7am to 7pm) and given food and water ad libitum under a protocol approved by the Institutional Animal Care and Use Committee at Florida Atlantic University.

Tamoxifen treatment:

Tamoxifen (Sigma-Aldrich) was dissolved in corn oil at a concentration of 20mg/ml and stored at 4°C. Mice were injected with tamoxifen at a dose of 75mg/kg of body weight for five consecutive days.

Tissue collection and processing:

Seven days after the last tamoxifen injection, mice were euthanized by isoflurane overdose and cardially perfused with phosphate-buffered saline (PBS) followed by 4% paraformaldehyde (PFA). The brain and peripheral organs, including the spleen, salivary gland, submandibular gland, sublingual gland, pancreas, and submandibular lymph nodes, were removed and post-fixed in 4% PFA overnight. The tissues were cryoprotected in 20% sucrose solution in 1x PBS overnight and frozen over dry ice in optimal cutting temperature (OCT) compound (Tissue-Tek) just prior to sectioning. Tissue sections were cut at 40 μm thickness using a cryostat. Brain and spleen sections were cut free-floating placed in cryoprotectant (Ethylene glycol/polyethylene glycol in 0.1M PB) and stored at -20C until use. Pancreas and salivary glands were cut at 40 μm, directly placed on a SuperFrost Plus slide (ThermoFisher), and stored at -80C until use. Tissue sections were washed 3 x 5min in 1x PBS and then incubated for 10min in 0.7% sudan black, to decrease autofluorescence, prior to mounting on a slide and cover sipping with Prolong Gold Mounting medium.

Microscopy and image analysis:

Images of whole organ was conducted on a 5mmx5mm grid under USB Digital Microscope with Flexible Arm Observation Stand (2MP). Tissue sections were imaged using the BZ-X Series 710 All-in-One Fluorescence Microscope brightfield and epi-fluorescent microscope at 10x magnification. The TdTomato signal was detected using excitation at 561 nm and emission at 595-695 nm and transmitted light was used to determine organ structure. 20x collages of entire sections were stitched using the Keyence Image Viewer software. Transmitted light and fluorescent light channels were merged and scale bars were added using ImageJ software (NIH). Identification of TdTomato+ cell types for descriptive analysis used Amano et al 2012 (Amano et al., 2012) and Atlas of Human Histology A Guide to Microscopic Structure of Cells, Tissues and Organs (Sorenson & Brelje, 2014) for anatomical descriptions.
